# Multimodality Cardiac Imaging in COVID-19 Infection

**DOI:** 10.3390/medicina59071223

**Published:** 2023-06-29

**Authors:** Sebastian Militaru, Anca Mihu, Amelia Valentina Genunche-Dumitrescu, Carmen Daniela Neagoe, Taina Elena Avramescu, Octavian Istratoaie, Ioana-Andreea Gheonea, Cristian Militaru

**Affiliations:** 1 Craiova University of Medicine and Pharmacy, 200349 Craiova, Romania; 2 Department of cardiology, Emergency Clinical County Hospital of Craiova, 200642 Craiova, Romania; 3 Sport Medicine and Physiotherapy Faculty, University of Craiova, 200585 Craiova, Romania; 4 Cardiomed Clinic, 200513 Craiova, Romania

**Keywords:** COVID-19, myocarditis, multimodality imaging, echocardiography, cardiac magnetic resonance

## Abstract

COVID-19 infection often produces cardiovascular complications, which can range from mild to severe and influence the overall prognosis. Imaging is the cornerstone for diagnosing initial COVID-19 cardiovascular involvement as well as treatment guidance. In this review, we present the current state of the literature on this subject while also emphasizing possible algorithms for indicating and executing these investigations.

## 1. Introduction

Coronavirus disease 2019 (COVID-19) is caused by severe acute respiratory syndrome coronavirus 2 (SARS-CoV-2) and has become a worldwide pandemic in a short time, having numerous socioeconomic effects [1]. In Romania, during the period of March 2020–2022, the total number of cases of SARS-CoV-2 infection exceeded 3.3 million, and over 67,000 deaths were recorded. The most common manifestation of the disease is acute pneumonia, which can evolve toward respiratory distress syndrome, multiple organ failure, and death. Involvement of the cardiovascular system in critical patients with COVID-19 has proved to be frequent (19.7%) [2] and represents, together with cardiovascular risk factors and pre-existing cardiovascular pathology, a negative predictor of the unfavorable evolution of the disease. Although the mechanisms by which SARS-CoV-2 produces negative effects on the cardiovascular system are complex and incompletely elucidated, it has been shown that they are mostly the consequence of interaction with the angiotensin-converting enzyme 2 (ACE-2) receptor. Although it was initially considered that the administration of drugs that inhibit the renin–angiotensin–aldosterone system (angiotensin-converting enzyme inhibitors and aldosterone receptor blockers) could have unfavorable effects in terms of susceptibility in contacting the disease and progression towards unfavorable evolution, the results of the BRACE study presented at the European Congress of Cardiology 2020 demonstrated that the use of angiotensin receptor antagonists (ARAs) has no impact on clinical status or prognosis at 30 days; the study compared the demonstrated benefit of this class of drugs and the lack of clear evidence of worsening or precipitating SARS-CoV-2 [3].

The cardiovascular manifestations described in the context of COVID-19 are diverse, including acute coronary syndromes, myocarditis, venous thromboembolism, heart failure, or arrhythmias [4].The introduction of vaccination against SARS-CoV-2 had the effect of a considerable reduction in severe forms of the disease but raised questions that were related to the potential risk of post-vaccination myopericarditis in some cases.

Advanced cardiac imaging has an important role in both diagnosis and risk stratification in cases of patients with COVID-19 who have cardiovascular complications [5]. The present article aims to describe the main imaging aspects of the secondary cardiovascular complications of COVID-19. This is not a systematic review, but instead an article aimed at reviewing the current literature, using the information and personal experiences detailed for hopefully enhancing the clinical practice, especially for those who perform cardiovascular imaging in COVID-19 patients.

## 2. Pathophysiology of Cardiovascular Complications Secondary to COVID-19

The cardiovascular complications produced by SARS-CoV-2 mainly refer to the coagulopathy of COVID-19 and myocardial injury, phenomena whose mechanisms of occurrence are not completely known until now. Coagulopathy occurs as a result of endothelial injury that is characterized by increased von Willebrand factor release, platelet activation, and hypercoagulability; moreover, the inflammatory status secondary to viral infection can cause hemostasis abnormalities similar to those described in sepsis: disseminated intravascular coagulopathy, which is manifested by increased concentrations of D-dimers and fibrinogen and by thrombocytopenia [6,7].

Myocardial injury is common in critical COVID-19 patients, and it is an independent predictor of mortality during hospitalization. Myocardial injury secondary to COVID-19 is characterized by an increase in markers of myocardial necrosis (troponin T, I, CK-MB, myoglobin) and natriuretic peptides (NT-proBNP) [8]. The pathophysiological mechanisms involved are not completely explored; currently, it is considered that both the hypothesis of the involvement of the direct mechanisms of viral replication at the level of cardiomyocytes in addition to indirect mechanisms, which occur as a result of the release of systemic cytokines, may be involved in the occurrence of myocardial injury in critical patients with COVID-19 [9].

The spectrum of cardiovascular complications secondary to COVID-19 is quite broad and may include acute coronary syndromes [10], myocarditis [11], left ventricular dysfunction, right ventricular dysfunction/acute cor pulmonale [12], Takotsubo cardiomyopathy, pericardial effusion, and arrhythmias [13,14,15]. For all of these cardiovascular complications, the first non-invasive imaging investigation is transthoracic echocardiography. These complications are summarized in Table 1.

Even if myocarditis is the most common manifestation of COVID-19 related cardiac injury, there have been increasing reports of Takotsubo syndrome in this population. Takotsubo syndrome, popularly called “broken heart” syndrome, is generally believed to be caused by extreme stress. Though the exact physiological pathway remains unknown, the underlying systemic stress and sheer numbers of infections caused by SARS-CoV-2 seem to be the factors for this increased prevalence during the COVID era [16,17,18].

## 3. Echocardiography

Echocardiography represents the first line of imaging diagnosis of cardiovascular complications of COVID-19, and it has the advantages of being a widely available, fast, portable, non-ionizing, relatively cheap, and reproducible method. Relatively close contact with the patient (thus increasing the infectious risk) is an important disadvantage, which is why specific measures regarding the examiner’s equipment are recommended, as well as the use of rapid protocols such as point-of-care ultrasound (POCUS) or focused cardiac ultrasound (FOCUS) to shorten the examination time [19]. The use of these protocols focuses on the evaluation of the size and systolic function of the LV (left ventricle) and RV (right ventricle), interventricular septal flattening, signs of pulmonary embolism and pulmonary hypertension, pericardial effusion, caliber of the inferior vena cava and inspiratory collapse, and on the monitoring of the evolution of the ultrasound parameters of the cardiac function [20].

The most common echocardiographic finding in patients with COVID-19 was represented by changes in the parameters of the right ventricle, with dilation occurring in 49% of cases and RV systolic dysfunction occurring in 40% of cases, according to an article published by Bonnemain et al. [21]. Changes in these parameters are closely related to the severity of pulmonary damage and are associated with the increase in cardiac biomarkers, prothrombotic status, and important inflammatory syndrome. In a significantly smaller percentage (about 10%), the presence of LV systolic dysfunction was observed [22], with some of the patients being known to have pre-existing ischemic coronary disease. RV systolic dysfunction was predominantly associated with a reduction in radial function; this was evaluated by the reducing fractional area change (FAC) in contrast to the preservation of a relatively normal longitudinal function, which was evaluated using tricuspid annular plane peak systolic excursion (TAPSE) [5]. In patients with RV systolic dysfunction, a significant percentage, approximately 20%, presented pulmonary embolism; this percentage could be underestimated considering that not all patients were evaluated using CT pulmonary angiography. In addition to the standard parameters for evaluating RV systolic function, a Chinese study conducted on 120 COVID-19 patients used advanced speckle tracking echocardiography techniques to identify RV longitudinal strain changes (RVLS). The results of the study demonstrated that RVLS was a strong predictor of mortality and was superior to FAC or TAPSE [23]. Another well researched area has been the changes in regional and global strains; one study looked at the strain data of 100 consecutive COVID-19 patients and showed altered RV free wall longitudinal strain in 40% of patients while also showing that poor clinical status was mostly corelated with worsening RV free wall strain in a pattern that was suggestive of cor pulmonale [24]. This particular finding has been repeatedly demonstrated is other studies [25] and begs the question of whether RV speckle tracking should be implemented in clinical routine when assessing COVID-19 patients. However, it is time-consuming, vendor-dependent, and image quality-dependent to a large degree.

**Figure 1:**
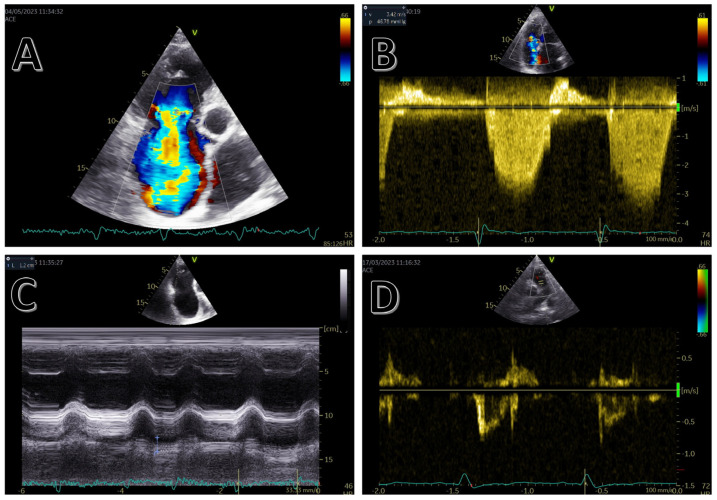
Echocardiographic markers of right ventricular impairment. (**A**) Significant tricuspid regurgitation. (**B**) High tricuspid gradient. (**C**) Right ventricular disfunction demonstrated by low TAPSE. (**D**) Short pulmonary acceleration time.

Regarding the impairment of the LV systolic function in critical COVID-19 patients, it was initially considered to be significant, though subsequent studies have proved it to be much less frequent and important compared with the RV impairment [26]. In most cases, hyperdynamic left ventricular ejection fraction (LVEF) was objectivized, but biventricular involvement was proven in certain studies; the results of such a study conducted in Turkey demonstrated a higher rate of biventricular dilatation and biventricular systolic dysfunction in patients with severe forms of COVID-19 compared with those that had mild forms [27]. In this case, the severity criteria of COVID-19 were considered to be a respiratory rate ≥ 30 breaths/minute, oxygen saturation ≤ 93% at rest, partial pressure of arterial oxygen, fractional concentration of inspired oxygen ≤ 300 mmHg, presence of critical complication (septic shock, multiple organ dysfunction/failure requiring ICU admission), or any type of respiratory failure that required mechanical ventilation. In an excellent metanalysis by Hothi et al., the authors compiled the data from several different studies on echographic cardiac involvement in COVID-19 patients vs. controls or a defined subgroup. The data showed that there is a consensus between all studies analyzed, demonstrating that the mainstay of COVID-19 cardiac involvement is RV impairment (Figure 1—large RV size, low TAPSE, low FAC, low tissue velocities and low longitudinal strain), while the LV size, LVEF, and longitudinal strain remain similar between groups [28].

On the other hand, as LV strain analysis has been a hot topic in cardiovascular imaging for the past 10 years, there has been extensive research into the role of LV longitudinal strain in COVID-19 patients. One such study included 81 consecutive COVID-19 patients, showing that there is actually a pattern of segmental longitudinal dysfunction in the basal segments [29]. This finding is reasonable, as we already know from CMR data that myocarditis damage is the largest in the basal part of the inferior wall, as well as the fact that the scar pattern in pulmonary hypertension is also located in the basal part of the inferior interventricular septum. Moreover, other studies have also shown a correlation between the LV global strain and mortality in COVID-19 patients [24].

## 4. Cardiac Magnetic Resonance (CMR)

**Figure 2:**
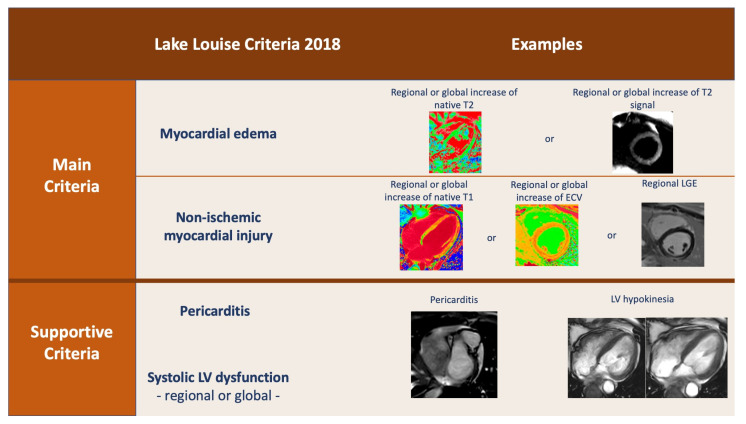
The Lake Louise criteria for diagnosis of myocarditis by cardiac magnetic resonance (reproduced after Ferreira et al.) [31].

Cardiac magnetic resonance has been established as the best non-invasive method for diagnosing myocarditis [30]. The Lake Louise criteria (Figure 2), which have been updated in 2018, clearly present the CMR changes that are characteristic to myocarditis.

The current main criteria include two categories: the first one is based in T2-weighted sequences, while the other is based on T1-weighted sequences [31]. The first one is mainly useful for the acute phases of myocarditis, while the second is used to show myocardial injury in the subacute/chronic phase [32].

On classic T2-weighted imaging (turbo spin-echo (TSE)-based, short tau inversion recovery [STIR] etc.), myocardial edema can be visually assessed on long-axis and short-axis images by using areas with hypersignalling compared with the rest of the myocardium [33]. A more advanced assessment can be performed on T2 mapping because individual native T2 recovery value can be calculated for each voxel, which has a more accurate evaluation of global or regional edema [34].

Myocardial injury can be demonstrated on late gadolinium enhancement (LGE) images and is conventionally classified as “fibrosis”. These magnetic resonance changes occur due to enlarged extracellular space and slow blood flow through a damaged myocardium. The typical aspect of myocarditis on LGE images is a spotty or linear hypersignal in the epicardium or mid-myocardic layers with obvious non-coronary distribution. In other pathologies (e.g., myocardial infarction), late gadolinium enhancement is permanent, as it shows necrosis of the myocytes; however, in myocarditis, late enhancement can be partially reversible in the following 6–12 months; thus, follow-up CMR evaluation can be indicated in COVID-19 myocarditis to assess permanent damage [35,36].

**Figure 3:**
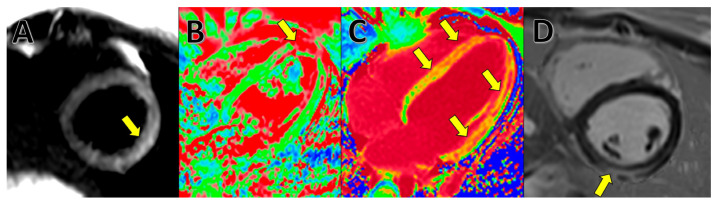
Typical myocarditis as seen on the CMR of a 16-year-old patient with COVID-19 infection. (**A**). Inferior apical wall T2 hypersignal on a short-axis plane. (**B**) High native T2 on a four-chamber T2 mapping sequence. (**C**) High native T1 on a four-chamber T1 mapping sequence. (**D**) Inferior wall late enhancement on the intramyocardial and epicardial layers of the inferior wall on a PSIR short-axis plane.

Native or enhanced T1 mapping can bring additional information and show diffuse “fibrosis”, edema, and enlarged extracellular volume. In many cases, the T1 value is increased in a much larger area than the late enhancement [37] (Figure 3).

There have been several large studies published in the last few years on the CMR changes in COVID-19 patients. One such study was the meta-analysis published by Kato et al. in 2022, which included 10,462 patients with COVID-19 infection that underwent CMR. While left ventricular ejection fraction and right ventricular ejection fraction were minimally lowered vs. the controls (by less than 3%), the presence of myocarditis was noted in 17.6% of patients along with various changes in different parameters (LGE in 27.5%, High T1 in 39.5%, High T2 in 38.1% of patients). These data further consolidate the importance of CMR in COVID-19 patients that exhibit any sign of myocardial damage [38].

Moreover, other studies have demonstrated an even higher proportion of cardiac involvement post-COVID-19 infection. One study examined 100 patients after a median of 77 days post-COVID-19 recovery using a comprehensive CMR protocol, which showed abnormal findings in 78 (78%) patients. Most frequently, native T1 and native T2 values were high, with the authors concluding that these two are in fact the parameters with the best discriminatory ability. Interestingly, of the 100 patients, only 33 had required hospitalization to recover, demonstrating that some myocardial damage is likely to occur, even in milder cases [39].

Addressing the topic of Takotsubo cardiomyopathy (TTC) in the MRI section is appropriate, because even though (TTC) is first suspected in echocardiography, cardiac MRI can differentiate between etiologies and establish a definitive diagnosis. TTC is characterized by the bulging of one of the left ventricular walls (most commonly the apex), which results in the dilemma of distinguishing between ischemic myocardial injury and TTC. Typically, the apical bulging is clearly visualized on long-axis cine images; however, the differential diagnosis is based on the tissue characterization sequences. Thus, in Takotsubo, there is normally no late gadolinium enhancement in the affected ventricular wall, but there is evidence of edema (hypersignal on T2-weighted images, high T2 values in T2 mapping, and possibly high T1 values in T1 mapping) [16,40].

Taking into account the literature as well as our personal experience, we have devised the following CMR protocol for COVID-19 patients (Table 2). The protocol was created with the purpose of having a complete exam with the shortest duration possible in order to reduce exposure. We achieved this by pushing the long-axis cine sequences to be observed after contrast injection and by skipping unnecessary steps.

## 5. Computed Tomography (CT)

CT is the main diagnostic tool for the assessment of pulmonary damage due to COVID-19 pneumonia [41]. Therefore, most COVID-19 patients will undergo a thoracic CT scan if they are admitted to a hospital.

**Figure 4:**
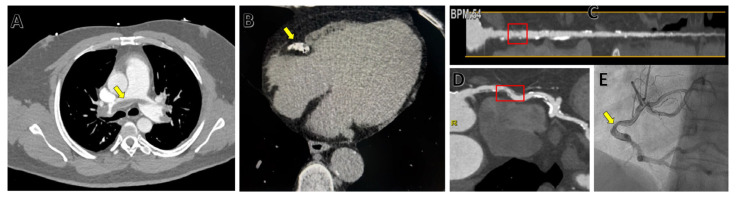
Examples of CT findings in COVID-19 patients. (**A**) Bilateral pulmonary artery embolism (yellow arrow) on an axial plane. (**B**) Large coronary calcified plaque on the right coronary artery (yellow arrow). (**C**) Straightened multiplanar reconstruction (MPR) of the right coronary artery showing moderate stenosis (60%–red box) in coronary CT angiography. (**D**) Curved multiplanar reconstruction (MPR) of the right coronary artery showing moderate stenosis (60%–red box) in coronary CT angiography. (**E**) Invasive coronary angiography confirming moderate stenosis (yellow arrow) of the right coronary artery. ((**B**–**E**) are for the same COVID-19 patient with chest pain, who also presented anomalous origin in the left anterior descending and circumflex arteries).

Moreover, CT is also recommended for evaluating cardiac complications of COVID-19 [42] as it can assess pulmonary embolism and coronary artery disease during the same scan (using a triple rule-out protocol) (Figure 4).

Another important factor in COVID-19 patients is the prevalence of high troponin levels. These have been associated with a worse prognosis due to myocardial damage [43,44,45]. In this context, coronary CT angiography is very important for excluding coronary artery disease in patients with raised levels of myocardial biomarkers, thus lowering the overall costs and complications of performing invasive coronary angiography.

Indeed, the guidelines indicate that cardiac CT is the preferred investigation method when in an intermediate coronary artery disease probability (11–89%) [46]. This is also postulated by the 2020 consensus paper published by Cosyns et al. on the imaging evaluation of COVID-19 patients with myocardial injury, where the authors stated that cardiac CT is the best tool for evaluating intermediate risk patients. However, this is probably not the case for patients with myocardial infarction where invasive coronary angiography is indicated, as studies have shown that a big portion of COVID-19-related myocardial infarctions are type 2 with no significant coronary artery obstruction [42].

Another important aspect of cardiac CT is also the evaluation of cardiovascular risk. To achieve this, the main application has been the coronary calcium score (CCS), which has been demonstrated by many large studies to be able to predict coronary events. In general terms, a coronary calcium score of more than 300–400 Agatston units is associated with a high risk of myocardial infarction in the next 5 years of life [47].

Taking all of the above information into consideration, we have formulated a protocol for CT angiography in COVID-19 patients that present chest pain and/or high troponin (without a clear STEMI pattern in their ECG), which is shown in Table 3.

The difficulty in acquiring a CT angiography of the coronary arteries as well as the pulmonary arteries lies in the delay between the opacification of the two artery groups (aorta/coronary opacification happens at least 10 s later than in the pulmonary arteries). Thus, for the protocol used, one must keep in mind that the total contrast media column should be around 20 s. We propose a manual breathhold command and start of acquisition for this exact reason while injecting enough contrast agent (e.g., 110 mL at 5.5 mL/s will be 20 s of uninterrupted contrast flow through the arteries).

## 6. Discussion

COVID-19 infection has probably affected more than a billion people worldwide over the past 4 years [48]. Of these people, a significant proportion may have some myocardial damage; the latest available studies and metanalyses estimate this number to fall between 15–40%. It is likely that the real percentage favors the lower end because of the many undiagnosed cases; however, it still represents a very high total number of cases [38,49,50].

Correct diagnosis of COVID-19-related cardiac injury due to myocarditis or myocardial infarction cannot be accomplished without multimodality cardiac imaging [5]. Indeed, from personal experience and from multiple studies, we know that high troponin levels are quite common in patients that present COVID pneumonia, whose presence influences outcomes. Therefore, it is important to find the cause of this myocyte injury [9,51].

While echocardiography continues to be the cornerstone of cardiac imaging, the role of cardiac CT to exclude coronary artery disease has been demonstrated to be beneficial and cost-effective in this population [42].

Moreover, the importance of CMR in the diagnosis of general myocarditis and particularly in COVID-19 myocarditis has been studied in detail. By using CMR analyses, we can quantify volumes and function better than any other modality, and by using the different available sequences, we can observe edema (T2-weighted, T1 mapping, T2 mapping) in the acute phase and a lasting scar in the long term (LGE) [19,28,38].

Moreover, CMR is also essential for differentiating between myocarditis and other possible cardiac manifestations of COVID-19, namely ischemic coronary disease and Takotsubo cardiomyopathy. Here, the presence and pattern of late gadolinium enhancement is essential, along with diffuse myocardial involvement such as edema and fibrosis [52].

Another important topic that must be addressed is the burden of COVID-19 infection on healthcare personnel. Of the staff performing cardiovascular imaging, it stands to reason that the most exposed would be the ultrasound technicians and doctors as they spend the most time in direct proximity per COVID-19 patient, with echocardiography also being the most commonly performed modality. However, CT and MRI staff are also quite affected, as shown by several studies performed in radiology departments [53,54,55]. This should be reflected in the protocols used to investigate COVID-19 patients; additionally, imaging societies have provided clear instructions on how to perform these investigations to mitigate the risk for healthcare personnel [56,57].

**Figure 5:**
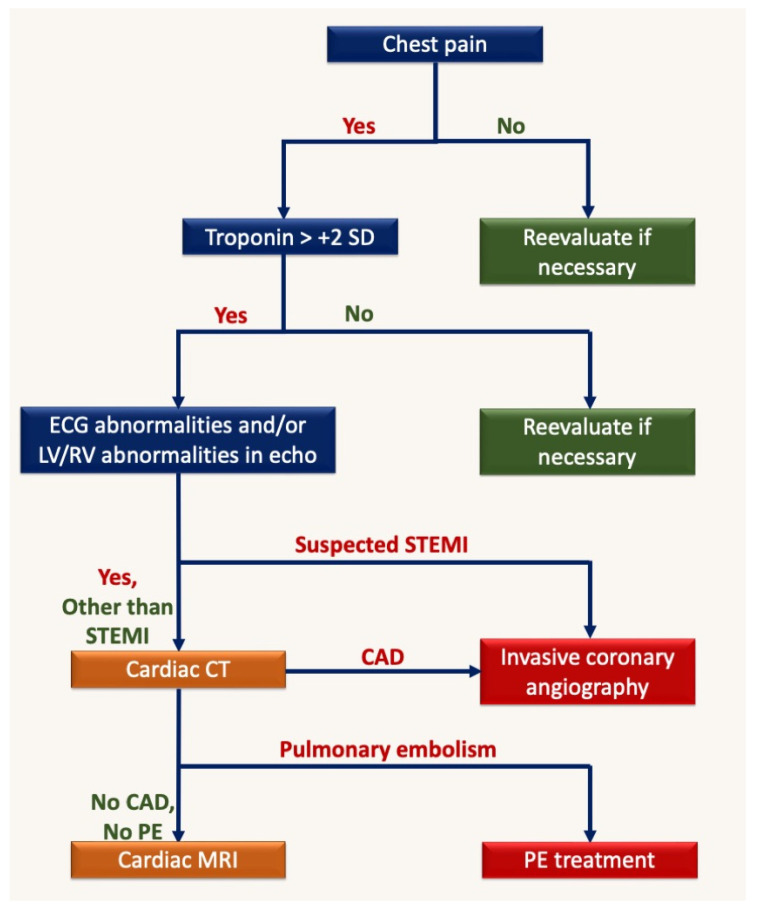
Imaging algorithm in confirmed COVID-19 patients with chest pain. SD, standard deviation; LV, left ventricle; RV, right ventricle; STEMI, ST-elevation myocardial infarction; CAD, coronary artery disease; PE, pulmonary embolism.

The following algorithm (Figure 5) tries to simplify the imaging approach for COVID-19 patients with chest pain. The main difference from the normal work-up of non-COVID patients is that cardiac CT should be performed with the double/triple rule-out protocol and should be performed in urgent care for all patients with positive troponin and ECG/echocardiography abnormalities without STEMI.

## 7. Conclusions

Cardiovascular involvement in COVID-19 infections represents a significant portion of the complications of this disease. As shown by numerous studies, cardiovascular imaging is essential for diagnosing and guiding treatment in such cases. Drawing from the literature and personal experience, we have reviewed the main uses for echocardiography, cardiac CT, and cardiac MR in COVID-19 patients while formulating potential practical approaches for performing and indicating the suggested use of these investigations.

## Figures and Tables

**Table 1:** Cardiac complication according to the stage of disease (adapted after A. Palmisano et al.) [5].

COVID-19 Phase	Cardiac Manifestations
Acute phase From symptom onset to symptom resolution	RV dysfunction: pulmonary hypertension
	Type I myocadiac infarction
	Type II myocardial infarction
	Myocarditis, Pericarditis
	Takotsubo cardiomyopathy
	Arrhythmias
Post-acute phase 3–4 weeks after onset	Myocarditis, Pericardits
Vaccination	Microvascular ischemia and myocardial infarction
	Myocardits, pericarditis

**Table 2:** Proposed cardiac magnetic resonance protocol in COVID-19 patients.

Sequence	Parameters
Survey	Low-resolution 3-axis standard survey of the thorax
Axial survey	20–25 slice axial single-shot white blood/black blood survey of the whole thorax
False 2-chamber	1 slice, 8 mm thickness SSFP cine
Short-axis SSFP cine	12–15 slices, 8 mm thickness, no slice gap
T1 mapping	3 slices, 8 mm slice thickness, 10–15 mm slice gap
T2 mapping	3 slices, 8 mm slice thickness, 10–15 mm slice gap
T2-weighted short-axis	TSE or STIR: 8 slices, 8 mm slice thickness, 0–5 mm slice gap
T2-weighted long-axis	TSE or STIR: 1 slice, 8
Contrast injection	0.15–0.2 mmol/kg, 3–4 mL/s
4-chamber SSFP cine	1 slice, 8 mm thickness
3-chamber SSFP cine	1 slice, 8 mm thickness
2-chamber SSFP cine	1 slice, 8 mm thickness
Right ventricle 2-chamber SSFP cine	1 slice, 8 mm thickness
LookLocker (MOLLI)	1 slice, 8 mm thickness
Short axis PSIR	10 slices, 8 mm slice thickness, 0–2 mm slice gap
4-chamber PSIR	1 slice, 8 mm thickness
3-chamber PSIR	1 slice, 8 mm thickness
2-chamber PSIR	1 slice, 8 mm thickness
Enhanced T1-mapping	3 slices, 8 mm slice thickness, 10–15 mm slice gap

**Table 3:** Proposed CT angiography protocol (for the ruling out of coronary artery disease and pulmonary embolism).

Phase	Details
Preparation	Administer B-blockers until heart rate is <70 bpm (not appliable for the newest machines with 256+ slices)
	Use venous canula of at least 20 G
Coronary Calcium Score	ECG-gated, breathhold
	Acquisition from tracheal carina until 2 cm under cardiac shadow on survey scans
	Calcium score calculation using dedicated software
CT Angiography	Injection protocol
	Use contrast agent with concentration of 300–400 mg of iodine/mL Two phase injection: 1. 80–120 mL of contrast media, 5–6 mL/s 2. 30–40 mL of saline, 5–6 mL/s
	ECG-gated, breathhold
	Acquisition from just over the aortic arch until 2 cm under the heart
	Acquisition window 30–80% Contrast monitor plane on the tracheal carina
	Manual breathhold and acquisition start: 1. Breathhold command when pulmonary artery has maximum opacification 2. Start 3 s delay acquisition when aorta starts opacifying

## Data Availability

Not applicable.
